# Stramonium as a Remedy in Dysmenorrhœa

**Published:** 1856-03

**Authors:** C. W. Ashby

**Affiliations:** Alexandria, Virginia


### ARTICLE II.

*Stramonium, as a remedy in Dysmenorrhœa.* By C. W. Ashby, M. D., Alexandria, Virginia.

Dysmenorrhœa has justly been regarded as one of the most painful diseases to which the female is liable. Its treatment, with the ordinary remedies of the books, has been so very unsatisfactory, that many practitioners, and indeed, some of our most able writers, consider it as incurable. Thus, many an unfortunate victim of this distressing malady, has been doomed to a miserable existence.

It was my privilege to sit under the teachings of the distinguished Dewees, and his warm and enthusiastic manner, inspired me with the most entire confidence in his Vol. Tinct. Guaicum. For the first seven years of my professional life, I prescribed this remedy for Dysmenorrhœa, in quite a large number of cases, but painful, though, the confession be, I am constrained to say, without the slightest benefit in a single case, so far as I know or believe. If the experience of the profession, generally, with this article, had not been similar to that of my own, I would have attributed my failures to a want of skill in the use of it. Other vaunted remedies in this disease, having met a like fate with Guaicum, it may be well supposed, that it is with great diffidence and distrust, that I hazard an opinion in favor of Stramonium, and ask for it a trial, and the calm adjudication of an enlightened profession.

I claim no originality in the use of this article, and have no preconceived opinions to sustain, or theories to advocate.

As far back as 1840, I first learned the use of Stramonium, and since then, in some fifteen or twenty cases, my success with it has been so remarkable, as I think, that an ardent desire to contribute something to the amelioration of suffering humanity alone, prompts me to make this communication. It is not my purpose to attempt to write a systematic treatise on Dysmenorrhœa, but rather to confine myself to the presentation of such *practical* views as may give a clear idea of the cases to which I think Stramonium is specially applicable.

1. The following case may serve to illustrate my subject better than any mere description: Mrs. Mary Jeffries, aged 27 years, a large, fat and healthy looking lady, informed me that she suffered intense pain in her back, hips and loins, at every monthly period; and that this had been the case, with more or less severity, from the time of her first catamenial flow. She thought she took cold at that time. She had always been remarkably regular at her month, being able to predict, with the greatest precision, the period for the commencement of her suffering; and although she did not remember ever having passed a month without pain, she thought her agony of late was more and more intolerable. The discharge generally continued three or four days, and was about the usual quantity and quality, in appearance, except that *invariably membranous lumps* were discharged. At, or about her periods, she had more or less uneasiness; swelling and pain of the mammary glands. The stomach and bowels were in good condition, and she regarded her general health as being very good, and complained only of slight lassitude and debility for several days after each attack. She had been married nine years, but had never conceived. Mrs. J. had been treated by several physicians, and had been, for several years, under the care of my friend and preceptor, Dr. Brodie S. Herndon. Dr. H. was then, as he is now, justly regarded as a highly cultivated and accomplished physician; and such was my entire confidence in his skill, as well as my own want of success, in the treatment of this disease, that I felt bound to inform the husband of the lady, that I could not promise her any permanent relief. She had taken various drugs, usually prescribed in such cases, but especially Dewees' Vol. Tinct. Guaicum, for a long continued period, and as she thought, without the slightest benefit. About this time she heard of the success of Dr. Sears of Flinthill, Va., in the treatment of similar cases; and after getting my consent, the husband went to see him. He sent her a prescription which had a most happy effect. At the first period, after commencing the treatment, she had far less pain than usual, and at the second, was entirely relieved. To the gratification of all parties, in a short time thereafter, unmistakable signs of pregnancy were manifested. For some years she enjoyed good health, and I was with her at three separate births, at full term; but ultimately her nervous system became very much deranged, and, after a protracted illness, she died of obscure disease of the lungs.

I soon learned from my friend, Dr. Sears, that it was the extract of Stramonium he had sent Mrs. J. He said that "his success, in quite a large number of cases, had been so uniform, that he now prescribed Ext. Stramonium in Dysmenorrhœa, with as much confidence as he did Quinine in Intermittent Fever." He referred me, as his authority, to Dr. Eberle's Practice, Vol. II., Article Dysmen, p. 529, and as I have not been able to find, in any regular treatise on diseases of females, nor indeed, anywhere else, the slightest notice of Stramonium in connection with this disease, I must be allowed to introduce the following extract. Dr. Eberle, after remarking that he had "used Dewees' Vol. Tinct. Guaicum with very indifferent success," says: "The remedy with which I have most frequently succeeded in effecting a cure, is the Ext. Stramonium. It is now about six years since I first resorted to this article for the cure of this affection; and I have on record, a considerable number of cases that yielded to its powers. My mode of employing it is, to give the one-eighth of a grain of the Ext. (Clutterbuck's preparation,) three times daily—commencing about four days before the expected return of the attack. I am persuaded, from what I have witnessed of its powers in this way, that we possess *no other article* which can, at all, be compared to it, as a remedy in this affection. Immediately previous to commencing with its use, the bowels should be freely opened by a purgative, and the patient ought to abstain from all kinds of stimulating food and drinks."

Under these circumstances, and with this high authority, I was eager to test for myself, this remedy. It was not very long before the following case was presented:

2. Miss Y., aged 23 years, had suffered pain every month, from the time of her first catamenial period—had gotten her feet wet at that time, and had the discharge suddenly checked, as she thought. From this time, her health had not been very good, but for several years past, she had been gradually getting worse; so, that at the time I saw her, she was truly an object of commiseration. She was a thin, pale, dejected looking person, and of a nervous temperament. A short time before the appearance of the discharge, at each period, she would be taken, generally, with violent pain in the stomach, followed by nausea and vomiting, which would continue, with more or less severity, for a day or two, and then often she would be seized with the most violent hysterical paroxysms, and become insensible for hours, and even days. At other times her sufferings would be described as resembling labor pains. She was very regular at her time; the discharge continued three or four days, rather pale and scanty. She had some uneasiness of the mammary glands, generally, about this time, and always discharged membranous lumps.

She had been the patient of several physicians, two of whom I knew to be men of large experience and reputation. Among other *remedies*, she had taken, *perseveringly*, large quantities of Vol. Tinct. Guaic. Just previous to my seeing her, she had been under the treatment of a Thompsonian doctor, by whom she thought she had been very much injured. Much notoriety had thus been given to this case, and I very naturally felt no little anxiety as to the result. I must say, that I have never seen before, or since, a more violent, and apparently, more hopeless case of this disease.

My first effort was, during the intervals of the attack, to improve the condition of the general system, by tonics and mild aperients. I gave the Stramonium as directed by Dr. Eberle, and also, Camphor and Morphine, in full doses, to palliate the pain, at the time of the attack. Not much decided relief was experienced at the first period. The same treatment was persevered in, except that the Stramonium was increased, so that the patient took from one-fourth to half a grain three times a day, and four times a day during the attack. I had the pleasure of learning, that at the next period she was greatly relieved; and in the course of three or four months she had entirely recovered. She subsequently married and became a mother.

3. I was consulted several years since, by a lady from Ohio, whilst on a visit to her mother, in Virginia. She had been married eight or nine years, without issue. She had always been very regular at her month, but suffered great pain, and discharged membranous lumps. She suffered a great deal with sick headache, and a nervous affection of the eyes, which was greatly aggravated at each monthly attack. She, at times, had a distressing cough, which, I was satisfied, was of a nervous character. All, together, life to her, was not life, but rather, the dragging out ot a miserable existence. She had been treated by various physicians, in several different places where she had lived; and various opinions had been expressed as to the character of her disease, and the cause of her sufferings. I wrote a prescription for this lady, advising in the intervals, on her return home, a general tonic course, cold bath, iron, &c., and the Stramonium to be commenced a week or ten days before the expected period, and also the purgative, (aloes, rhubarb and blue mass, 5 grs. each, I usually prescribe,) three days before the attack. If relief was not had, she was directed to increase the dose of the Stramonium gradually, until its decided effects upon the eyes were manifested. I also directed the lady to get in bed so soon as she felt the pain, apply flannels wrung out of hot water, over the region of the womb, and full doses of camphor and morphine every half hour until relief is had, and at the same time giving the Stramonium four or five times a day, during the attack. I had the pleasure of hearing that this lady's health was greatly improved by the prescription, and shortly afterwards she became pregnant.

I have selected these cases, though somewhat extraordinary in point of severity, because I regard them as well marked cases of Dysmenorrhœa, and having been treated unsuccessfully by the ordinary remedies, the efficacy ot Stramonium is thus, I think, more clearly exhibited.

One or two other cases I desire to serve as a type of a much larger class of cases, to which Stramonium is applicable.

4. Miss A., 19 years old, suffered severe pain, for two or three days at every monthly period. She was very regular, and the quantity and quality of the discharge natural, except that membranous lumps were constantly present. She looked well and insisted that her health was good, and had been unwilling that her mother should consult a physician until recently. During the intervals of the attack, she had been gay and cheerful, but recently a marked change in this respect, had been observed. I advised the Stramonium, purgative, anodynes, &c., to be used as directed in the last case; and in a short time was informed that her health was entirely restored.

5. I was informed that a servant woman was in the habit of losing three or four days from her work every month. Except at this time, she was able to do her work with entire satisfaction. She had been married for several years, without issue. I ascertained that it was a well marked case of membranous Dysmenorrhœa. I ordered the Stram., Anodynes, purgative and hot applications, (as is *now* my usual plan,) and in a few months, was informed that she had recovered, and was pregnant.

I might present a number of other cases similar to these, treated successfully in the same way; but as I design being brief, I prefer introducing a case which illustrates the circumstances under which Stramonium is not applicable.

6. Mrs. F., aged twenty-six, has suffered some derangement of her catamenia since her first womanhood. She was not regular; but sometimes the discharge would be one or two weeks too soon, and at other times, she would be five or six weeks without their return. She generally suffered pain, but not always, at her periods. Sometimes the discharge would be pale and scanty, then again, very profuse. She frequently discharged lumps, but not invariably. Some Leucorrhœa, but not a great deal, was usual with her. For several years past, her sufferings at her month have greatly increased, and though she is a fat, healthy looking woman, she is rarely without some pain, especially when she walks. I prescribed Stramonium, &c., for this lady, without seeing her, upon the representation of her husband. She used the remedies, perseveringly, for some time, but without the slightest benefit.

At first, I prescribed Stramonium in quite a number of cases similar to this, with similar results; and truth and honesty require that I should state, that a few cases also, have occurred in my practice, where I prescribed Stramonium with entire confidence, in which it has utterly failed.

*Remarks.*—1. Many practitioners, I am satisfied, have been misled by the name of this disease. Dysmenorrhœa, we all know, means a painful or difficult menstruation; but every woman who suffers pain, with a bloody discharge, has not the disease for which I recommend Stramonium. My sixth and last case, was clearly one of organic disease of the uterus—engorgement of the neck; and the pain was produced by the discharge of lumps of coagulable blood.

*Membranous Menstruation, as a title*, would far better designate the form of disease to which this paper has reference.

2. Membranous Menstruation, I think, is a disease of the ovaria, and although the nervous system *alone* is generally involved, yet sometimes, organic disease of the uterus, as an effect, occurs, and must be treated, before success can be hoped for from Stramonium. Barrenness and the painful discharge of membranous lumps are the characteristics of this disease. In this affection the mammary glands usually sympathise, and become swollen and painful, which is not the case in mere organic disease of the womb.

3. I once mentioned my treatment of Dysmenorrhœa with Stramonium, to Dr. Meigs, the distinguished Professor of Jefferson Medical College; he instantly replied, that it could act *only* as an Anodyne. With all deference to the learned Professor, I must say, that I think, this can not be true. I have not the slightest confidence in it as an Anodyne, and therefore invariably prescribe, in this disease, camphor and opium, as a palliative. If it acts only as an Anodyne, why were not the cases detailed, and others I might relate, relieved by the Anodynes, (far more potent than Stramonium,) which had been used for years? The modus operandi of most of our medicines, as yet, is involved in great mystery, and I shall not venture an opinion as to the mode of action of Stramonium, but I will say, *now* that we *know* that it exerts a specific influence in dilating the pupils of the eye, it does not require a great deal of credulity to believe that it may act by exerting a specific influence in dilating the Fallopian tubes, and even the aperture of the neck of the uterus.

4. It will be observed that I use much larger doses of Stramonium than directed by Dr. Eberle, and also, that in some cases, I commence the use of it a longer period before the expected attack. I have found it a very difficult matter to get a good article of Extract of Stramonium, probably owing to various causes connected with its manufacture. Also, I think, Clutterbuck's preparation, which he used, is much stronger than the extract of the shops, of the present day. To get the system gradually under the influence of the article, I have thought it best to take a little more time than Eberle directs to do it. I increase the quantity until the specific influence of the article on the pupils is observed, and I have never seen the slightest injurious effects resulting from its use, though I have prescribed it frequently, for fifteen years.

5. It has been justly said by a celebrated writer, that "all science is enriched *alone* by facts." I am fully aware of the power of "vis medicatrix naturæ," and that spontaneous cures not unfrequently occur in this, as in other diseases, and therefore have written under a painful sense of the uncertainty of medical facts. Professor Dewees announced his Vol. Tinct. Guaicum with so much emphasis and confidence, as to make the impression, that it was almost an infallible remedy. McIntosh, even more dogmatic, lauded his system of treatment with the bougie, as the only *rational* and *certain remedy*. It is to be regretted that these distinguished gentlemen committed the too common error of our profession, viz.: of recommending *too generally* and *too confidently* their peculiar remedy, without pointing out *accurately* the precise circumstances under which they used them. The high encomiums lavished upon these agents, have not been sustained by the experience of the profession; and they have been well nigh abandoned. Valuable remedies have thus, doubtless, sometimes, gone into disrepute. In a few cases of local obstruction, I have tested the value of the bougie, and I can hardly believe that Professor Dewees could have been entirely deceived as to the efficacy of Guaicum. Dr. Eberle, in regard to Stramonium, I think, has committed the same error with these gentlemen, and it is probably from the same cause, it is now not known to the profession, generally, as a remedy in Dysmenorrhœa. It is for this reason, *mainly*, I have been induced to record my experience, and to *attempt* to present, clearly, the cases of Dysmenorrhœa in which, I think, I have derived valuable results from the use of Stramonium.

In conclusion, I may say, that if amid the disappointments and trials incident to professional life, the use of Stramonium, as I have recommended, shall cause any woman to suffer a single pang less, or afford any brother half the pleasant reminiscences it has afforded me, I shall be more than gratified, and amply repaid.

